# Cross-Cultural Adaptation and Psychometric Validation of the Cancer Survivors’ Unmet Needs Measure (CaSUN) in Russian- and Kazakh-Speaking Cancer Survivors in Kazakhstan

**DOI:** 10.3390/healthcare14183012

**Published:** 2026-09-14

**Authors:** Gulnar Karabasova, Kerbez Kimatova, Nurgul Abenova, Anar Tulyayeva, Zalika Klemenc-Ketiš, Perizat Aitmaganbet

**Affiliations:** 1 Department of Public Health and Healthcare with the Course of Nursing, West Kazakhstan Marat Ospanov Medical University, Aktobe 030000, Kazakhstan; perizat.a@zkmu.kz; 2 Department of General Practice No. 1, West Kazakhstan Marat Ospanov Medical University, Aktobe 030019, Kazakhstan; nurgul.abenova@gmail.com; 3 Department of Oncology, West Kazakhstan Marat Ospanov Medical University, Aktobe 030000, Kazakhstan; dekart_85@mail.ru; 4 Department of Family Medicine, Medical Faculty, University of Maribor, 2000 Maribor, Slovenia; zalika.klemenc@um.si; 5 Community Health Centre Ljubljana, 1000 Ljubljana, Slovenia; 6 Department of Family Medicine, Medical Faculty, University of Ljubljana, 1000 Ljubljana, Slovenia

**Keywords:** cancer survivorship, unmet supportive care needs, CaSUN, psychometric validation, cross-cultural adaptation, patient-reported measures, healthcare services

## Abstract

**Background/Objectives:** The growing population of cancer survivors has increased the need for reliable, culturally appropriate instruments to identify unmet supportive care needs. The Cancer Survivors’ Unmet Needs Measure (CaSUN) is a survivorship-specific assessment tool, but validated Russian and Kazakh versions have not previously been available. This study aimed to translate and culturally adapt CaSUN, evaluate its psychometric properties in cancer survivors in Kazakhstan, and examine unmet needs relevant to survivorship care. **Methods:** This cross-sectional psychometric validation study included 404 cancer survivors (Russian version, *n* = 201; Kazakh version, *n* = 203). Translation and cultural adaptation involved forward- and back-translation, expert review, cognitive debriefing, and pilot testing. Internal consistency was assessed using Cronbach’s alpha. Construct validity was evaluated using exploratory (EFA) and confirmatory factor analysis (CFA), with measurement invariance examined across language versions. Unmet needs and associated demographic and clinical factors were also examined. **Results:** Both versions demonstrated excellent internal consistency (Cronbach’s alpha: Russian = 0.966; Kazakh = 0.978). KMO values were 0.920 and 0.940, respectively (Bartlett’s tests, *p* < 0.001). EFA yielded a five-factor solution explaining 68.1% and 64.2% of the variance in the Russian and Kazakh versions, respectively; however, parallel analysis supported five factors in the Russian sample but suggested three in the Kazakh sample, indicating greater structural uncertainty for the Kazakh version. CFA demonstrated good incremental fit in both language groups (Russian: CFI = 0.975, TLI = 0.973; Kazakh: CFI = 0.973, TLI = 0.971), although RMSEA (0.096 and 0.102, respectively) and SRMR (0.116 and 0.099, respectively) indicated residual model misfit. Metric invariance was supported, while scalar invariance was supported by conventional fit index criteria but should be regarded as provisional. The most frequent unmet needs concerned medical care, up-to-date information, and involvement in health management. Younger age, male sex, urban residence, a history of cancer recurrence, and Kazakh language completion were independently associated with more unmet needs. **Conclusions:** The Russian and Kazakh CaSUN versions demonstrated high internal consistency and evidence supporting construct validity and cross-language comparability. These culturally adapted versions may support systematic needs assessment and patient-centered survivorship care in multilingual oncology services in Kazakhstan.

## 1. Introduction

Advances in cancer screening, diagnosis, and treatment have transformed cancer from an acute life-threatening disease into a chronic condition, and the global population of cancer survivors continues to grow, with an estimated 53.5 million people living for at least five years after a cancer diagnosis [1,2]. However, improved survival does not equate to complete recovery: many survivors experience persistent physical symptoms, psychological distress, fear of recurrence, social and occupational challenges, and financial toxicity, all of which can adversely affect quality of life [3,4,5]. These challenges are often insufficiently addressed by health systems and are commonly described as unmet supportive care needs [6], reflecting the gap between the support survivors require and the care they actually receive [7,8,9,10]. Systematic assessment of these needs is therefore essential for person-centered survivorship care, allowing vulnerable survivors to be identified and timely, targeted interventions to be provided.

Several instruments assess supportive care needs in oncology, but many were designed for patients undergoing active treatment rather than survivors beyond the acute phase [11,12]. The Cancer Survivors’ Unmet Needs Measure (CaSUN) was developed specifically for survivors and comprises 35 items across five conceptual domains: existential survivorship, comprehensive cancer care, information, quality of life, and relationships [13]. CaSUN has since been validated in several countries and is among the most widely applied survivorship-specific patient-reported outcome measures [14,15,16,17,18].

Despite its international use, validated Russian and Kazakh versions of CaSUN have not previously been available, creating an important gap for Kazakhstan and potentially for other multilingual settings in Central Asia. This gap carries particular weight given Kazakhstan’s bilingual legal and social fabric: under Article 7 of the Constitution of the Republic of Kazakhstan, Kazakh holds the status of the state language, while Russian is used on an equal footing within state institutions. The two languages, moreover, diverge not only administratively but linguistically and culturally—Kazakh belongs to the Turkic family and Russian to the Slavic—and the two speech communities differ in the norms governing disclosure of personal difficulty. Foremost among these is uyat, a locally resonant norm of propriety and restraint shown to discourage open discussion of sensitive personal, family, or, in documented cases of intimate partner violence, health-related concerns [19,20]. Where such norms shape how comfortably a respondent endorses an item addressing emotional, relational, or existential need, the equivalence of meaning across language versions cannot simply be presumed, it must be demonstrated. Kazakhstan is a bilingual country, and the absence of validated survivorship needs measures in both languages may limit equitable needs assessment, comparability with international evidence, and the integration of patient-reported information into routine oncology services. Evidence on supportive care needs after cancer treatment in Kazakhstan also remains limited. Two research questions therefore guided the present investigation. First, do the Russian and Kazakh CaSUN versions demonstrate adequate reliability and construct validity, and to what extent can scores derived from the two language versions be considered comparable? Second, which demographic and clinical characteristics are independently associated with the burden of unmet supportive-care needs among cancer survivors in Kazakhstan, and does this association extend to the language in which the instrument was administered? We hypothesized a priori that age, sex, place of residence, educational attainment, disease stage, recurrence status, and language of administration would each be independently associated with this burden. Accordingly, this study aimed to translate and culturally adapt CaSUN into Russian and Kazakh, evaluate the reliability, construct validity, and cross-language measurement equivalence of both versions, and describe the prevalence and determinants of unmet supportive care needs. By combining psychometric validation with an assessment of healthcare-related needs, the study was designed to provide evidence relevant to survivorship care planning and service development in a multilingual healthcare system.

## 2. Materials and Methods

### 2.1. Study Design, Setting, Participants, and Ethics

This study was approved by the Local Bioethics Committee of the West Kazakhstan Marat Ospanov Medical University (protocol 2, 28 February 2025; registration number 2-2025/059-D) and was conducted in accordance with the Declaration of Helsinki. All participants provided written informed consent, and data were processed anonymously.

Kazakh is the state language of Kazakhstan, while Russian remains widely used as a second language, reflecting the country’s history as part of the Soviet Union. CaSUN was therefore translated and validated in both languages to ensure accessibility across the population. Language of instrument administration was based on each participant’s self-reported preferred language of communication rather than random assignment.

Data were collected between 1 May and 31 December 2025, at the Medical Center of West Kazakhstan Marat Ospanov Medical University (Aktobe, Kazakhstan). Eligible patients were invited by investigators to participate during outpatient visits to the Medical Center’s polyclinic. Inclusion criteria were: age 18 years or older; a confirmed diagnosis of malignant neoplasm; completion of primary treatment (surgery, chemotherapy, and/or radiotherapy) between 6 months and 5 years before enrolment; and the ability to complete the questionnaire independently or with interviewer assistance. Exclusion criteria were: terminal-stage disease, active disease recurrence or progression at the time of enrolment, and severe cognitive impairment or psychiatric illness precluding informed participation. A history of cancer recurrence did not preclude participation provided that there was no active disease recurrence or progression at the time of enrolment. Investigators explained the study aims, its voluntary nature, and the questionnaire procedure to all patients. Of 457 eligible patients invited, 404 agreed to participate (53 declined).

### 2.2. Instrument and Cross-Cultural Adaptation

CaSUN, developed by Hodgkinson and colleagues, measures unmet supportive care needs in cancer survivors [13]. It comprises 35 items across five domains—existential survivorship, comprehensive cancer care, information, quality of life, and relationships—plus six positive-change items and one open-ended question on positive consequences of the cancer experience. Items were scored per the original CaSUN manual; higher scores indicate greater unmet need, and domain-level reliability was calculated according to the original CaSUN structure. Permission to translate and use the questionnaire was obtained from the original developers, and translation and cultural adaptation were conducted separately for the Russian and Kazakh versions following internationally accepted guidelines for cross-cultural adaptation of patient-reported outcome measures [21,22].

Translation and cultural adaptation were conducted between 1 March and 30 April 2025, following a structured process comprising independent forward translation by two bilingual experts, reconciliation of the resulting versions, back-translation by two independent translators blinded to the original instrument, and cognitive debriefing interviews. Throughout this process, a multidisciplinary expert panel—comprising oncologists, public health specialists, psychologists, and linguists with relevant professional qualifications in oncology, translation studies, psychology, and linguistics—reviewed all versions to evaluate semantic, conceptual, and cultural equivalence, consistent with established guidelines for the cross-cultural adaptation of patient-reported outcome measures [21,22]. Clarity of the resulting translations was subsequently assessed separately in Kazakh and Russian through pilot testing with 20 respondents per language, a sample considered adequate for identifying major comprehension problems and consistent with practice in comparable single-center adaptation studies, although a larger sample would have permitted more precise estimation of item-level clarity. The bilingual questionnaire was finalized following revisions informed by the pilot results.

### 2.3. Statistical Analysis

Statistical analyses were performed using R software (version 4.5.2). Descriptive statistics were used to summarize participant characteristics and questionnaire responses. Continuous variables were presented as means and standard deviations or medians and interquartile ranges, while categorical variables were presented as frequencies and percentages.

Internal consistency reliability was assessed using Cronbach’s alpha coefficients for the total scale and individual domains. Values ≥ 0.70 were considered acceptable, ≥0.80 good, and ≥0.90 excellent.

Construct validity was evaluated using exploratory factor analysis (EFA). Prior to factor extraction, sampling adequacy was assessed using the Kaiser–Meyer–Olkin (KMO) measure and Bartlett’s test of sphericity. Factors were extracted using principal axis factoring with Promax rotation. The number of retained factors was determined using parallel analysis, scree plot inspection, eigenvalues, and theoretical interpretability. Factor loadings ≥ 0.30 were considered meaningful.

Confirmatory factor analysis (CFA) was performed to evaluate the fit of the theoretical five-factor model. Model fit was assessed using the Comparative Fit Index (CFI), Tucker–Lewis Index (TLI), Root Mean Square Error of Approximation (RMSEA), Standardized Root Mean Square Residual (SRMR), and χ^2^/df ratio. Because fit indices may provide different information about model adequacy, they were interpreted jointly rather than on the basis of a single cutoff. Measurement invariance across the Russian and Kazakh language groups was subsequently evaluated using sequential configural, metric, and scalar models. Changes in comparative fit indices between increasingly constrained models were considered alongside the absolute fit indices when assessing invariance.

Because CaSUN items are ordered-categorical, both the exploratory analysis and the confirmatory and invariance analyses described above were conducted on polychoric correlation matrices, with the latter two specifically estimated using the WLSMV estimator appropriate to ordinal indicators. We further note that the exploratory and confirmatory analyses draw on the same sample. Because EFA capitalizes on sample-specific associations, this precludes independent confirmation of the resulting structure. To partly offset this, the confirmatory analysis prioritized the prespecified five-factor CaSUN model [13], rather than a structure derived post hoc from the exploratory results in the same data; independent replication of this factor structure in a separate sample remains an important direction for future research.

The ordinal continuum represented by the CaSUN response categories is interpreted as the degree of unresolved supportive-care need burden, rather than need severity per se. Under this interpretation, “met need” is treated as intermediate between “no need” and “unmet need” not because it carries residual severity, but because it reflects the prior occurrence of a need-domain concern—a status that “no need” does not share—while carrying no continuing unresolved burden, consistent with the original CaSUN scoring rationale [13].

Frequencies of unmet supportive care needs and positive change items were analyzed descriptively. The number of unmet needs was calculated by summing all items coded as 2 or 3 across the 35 CaSUN unmet needs items. Multivariable linear regression analysis was performed to identify demographic and clinical factors independently associated with unmet supportive care needs. Variables entered into the model included age, sex, place of residence, education level, cancer stage, recurrence status, and questionnaire language version. These variables were selected based on their established or plausible association with unmet supportive-care needs in the prior CaSUN and cancer survivorship literature. Statistical significance was defined as *p* < 0.05. One participant with unclassified (‘other’) education level was excluded from the multivariable regression model due to insufficient category size (final regression *n* = 403). Analyses used the readxl, dplyr, psych (with GPArotation), lavaan, semTools, broom, and openxlsx packages.

## 3. Results

### 3.1. Participant Characteristics

A total of 404 cancer survivors participated in the study, including 201 respondents who completed the Russian language version of the questionnaire and 203 who completed the Kazakh language version. The mean age of participants was 60.7 years (SD 9.8; range 24–87 years). Women comprised 68.3% (*n* = 276) of the sample, while 31.7% (*n* = 128) were men. Most participants resided in urban areas (69.8%, *n* = 282), whereas 30.2% (*n* = 122) lived in rural settings.

Regarding educational attainment, 49.0% (*n* = 198) had vocational education, 27.0% (*n* = 109) had higher education, and 23.8% (*n* = 96) had secondary education. Stage II disease was the most frequently reported stage (57.4%, *n* = 232), followed by stage I (17.6%, *n* = 71), stage III (10.9%, *n* = 44), stage IV (3.0%, *n* = 12), and unknown stage (11.1%, *n* = 45). A history of cancer recurrence was reported by 19.8% (*n* = 80) of participants, while 65.8% (*n* = 266) reported no recurrence and 14.4% (*n* = 58) were uncertain of their recurrence status.

The most common cancer diagnoses were kidney cancer (29.0%, *n* = 117), breast cancer (26.5%, *n* = 107), cervical cancer (10.4%, *n* = 42), and gastric cancer (7.2%, *n* = 29) (Table 1). Because language of administration was not randomly assigned, Russian and Kazakh language groups were compared using independent-samples *t*-tests (age) and chi-squared tests (categorical variables). The groups did not differ significantly in age, sex, or residence (all *p* > 0.05), but differed significantly in education, marital status, employment, diagnosis, stage, and recurrence status (all *p* < 0.05; Table 1).

### 3.2. Reliability and Construct Validity

Both language versions demonstrated excellent internal consistency. Cronbach’s alpha for the total scale was 0.966 for the Russian version and 0.978 for the Kazakh version. Domain-specific alpha coefficients ranged from 0.905 to 0.948 in the Russian version and from 0.909 to 0.959 in the Kazakh version, indicating excellent reliability across all domains (Table 2).

Sampling adequacy for exploratory factor analysis was excellent for both versions (KMO = 0.920 for Russian and KMO = 0.940 for Kazakh), while Bartlett’s tests of sphericity were highly significant (*p* < 0.001), confirming the suitability of the data for factor analysis. EFA identified a five-factor solution in each language version, explaining 68.1% of total variance in the Russian version and 64.2% in the Kazakh version (Table 2). Parallel analysis using polychoric correlations supported the five-factor solution in the Russian sample but suggested only three factors in the Kazakh sample, indicating potential instability of the five-factor structure specifically in the Kazakh language version. Full EFA results (communalities, complete loading matrices, cross-loadings, and factor correlations) are reported in Tables S1–S3 of the Supplementary Materials.

Category frequencies for all 35 items are reported by language in Supplementary Table S6. The Kazakh sample showed no meaningfully sparse cells (minimum *n* = 5, item 13 only); the Russian sample showed sparser strong-unmet-need cells for a subset of items, most notably item 27 (*n* = 1). As a further sensitivity analysis, all items were recoded into a binary indicator (no need/met need = 0; weak/strong unmet need = 1) and the confirmatory model re-estimated separately by language. Standardized loadings were highly consistent with the primary four-category model (mean absolute difference: Russian = 0.057, Kazakh = 0.034), and the five-factor structure was unchanged. The largest discrepancies occurred at items with the sparsest category-3 cells identified above (Russian item 27: Δ = 0.205), consistent with reduced estimation precision for these specific items rather than a broader structural problem.

Despite this divergence, the overall solution remained broadly aligned with the conceptual organization of CaSUN, although the item clustering also indicated context-specific differences, particularly for practical and financial concerns.

Confirmatory factor analysis of the prespecified five-factor structure, estimated separately for each language using an estimator suited to the ordinal response format (WLSMV), yielded a broadly consistent picture across versions: comparative fit was good in both (Russian: CFI = 0.975, TLI = 0.973; Kazakh: CFI = 0.973, TLI = 0.971), while RMSEA and SRMR remained above conventional thresholds in each (Russian: RMSEA = 0.096, SRMR = 0.116; Kazakh: RMSEA = 0.102, SRMR = 0.099), indicating residual misfit that tempers, without negating, support for the five-factor model (Table 3). Standardized loadings, residual variances, and diagnostic warnings for each language are reported in full in Supplementary Tables S4 and S5, and the corresponding inter-factor correlations in Table 3b. Building on this corrected model, sequential invariance testing supported metric equivalence between the Russian and Kazakh versions (ΔCFI = +0.003, ΔRMSEA = −0.008; non-significant scaled χ^2^-difference test, *p* = 0.835) and, by conventional fit index criteria, scalar equivalence as well (ΔCFI = −0.003, ΔRMSEA = +0.003; Table 4)—though the latter is offered as a tentative rather than definitive conclusion, since the corresponding χ^2^-difference test could not be reliably computed, plausibly reflecting the item-level dependence discussed below. Comparisons of scores across the two language versions therefore appear reasonably, if not yet conclusively, warranted.

### 3.3. Unmet Supportive Care Needs

The mean number of unmet supportive care needs was 13.4 (SD 10.2), with a median of 13 needs (IQR 4–22) and a range from 0 to 35 unmet needs (Table 5).

**Figure 1:**
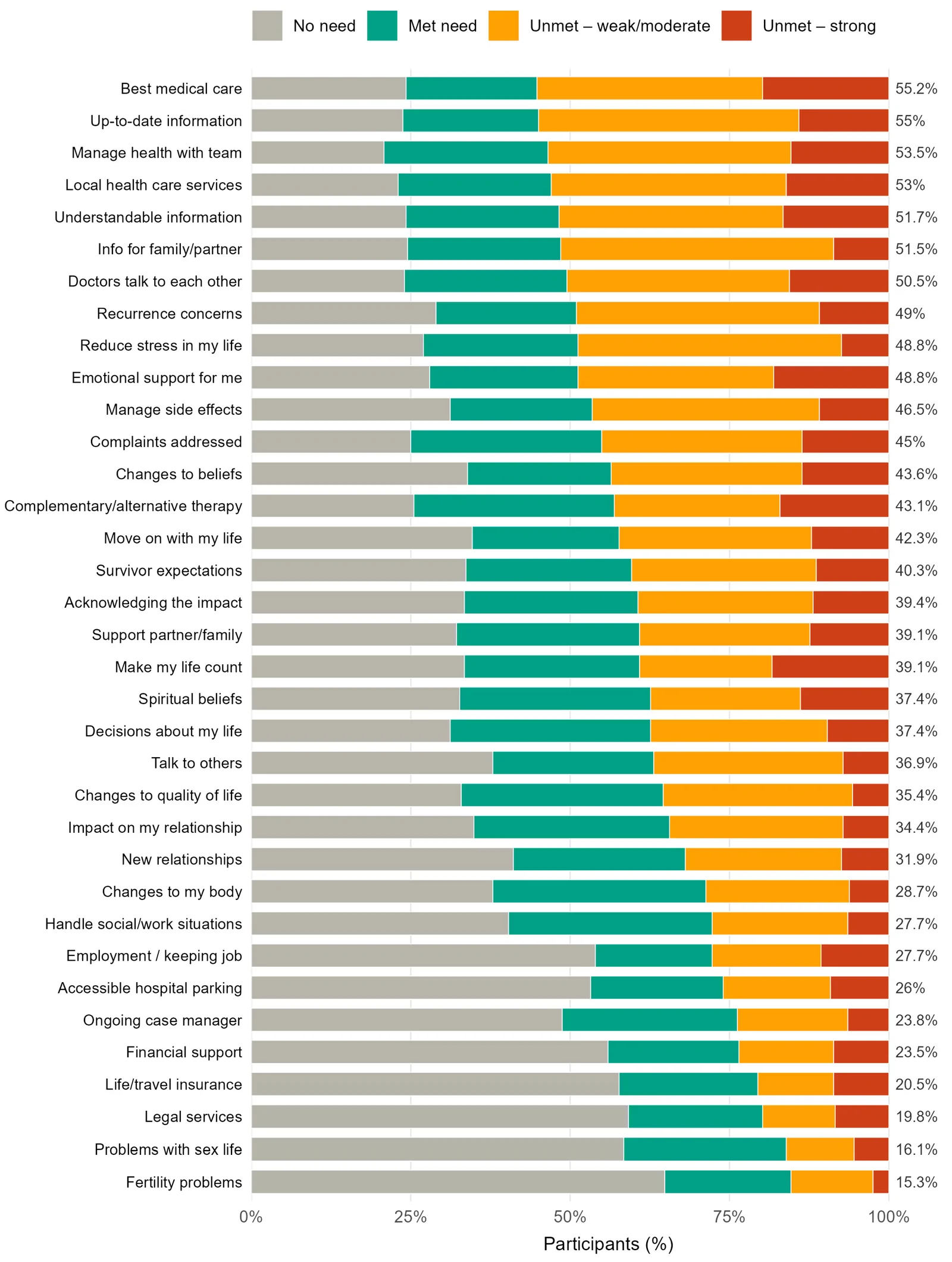
Distribution of responses across the 35 CaSUN items in the total study sample (*n* = 404). Percentages shown on the right represent the combined proportion of participants reporting weak/moderate or strong unmet needs. Items are ordered by the overall prevalence of unmet need.

The most frequently reported unmet needs were related to receiving the best possible medical care (55.2%, *n* = 223) and access to up-to-date information (55.0%, *n* = 222). Additional highly endorsed needs included managing health together with the healthcare team (53.5%, *n* = 216), access to local healthcare services when needed (53.0%, *n* = 214), information presented in an understandable format (51.7%, *n* = 209), information for family members and/or partners (51.5%, *n* = 208), and coordination between healthcare professionals (50.5%, *n* = 204). Psychosocial concerns were also prominent: 49.0% (*n* = 198) reported unmet needs related to fear of cancer recurrence, and approximately 48.8% reported needing help reducing stress in life (*n* = 197) or emotional support (*n*= 197). Twenty-two of the 35 items (63%) showed a statistically significant difference in prevalence between language groups, and on every such item the Kazakh language group reported the higher prevalence, most markedly for recurrence-related concerns, emotional support, managing side effects, and problems with sex life (Table 5). The full distribution of responses (no need, met need, and unmet need rated as weak/moderate or strong) for all 35 items is presented in Figure 1.

### 3.4. Factors Associated with Unmet Supportive Care Needs

Multivariable linear regression identified several independent predictors of unmet supportive care needs (Table 6). Younger age was significantly associated with a greater number of unmet needs (B = −0.221, 95% CI −0.305 to −0.137; *p* < 0.001). Female participants reported significantly fewer unmet needs than males (B = −5.08, 95% CI −6.82 to −3.35; *p* < 0.001). Place of residence was also independently associated with unmet needs. Rural residents reported significantly fewer unmet needs than urban residents (B = −7.89, 95% CI −9.76 to −6.01; *p* < 0.001). A history of cancer recurrence emerged as one of the strongest predictors. Participants without a history of recurrence reported substantially fewer unmet needs than those with a history of recurrence (B = −6.11, 95% CI −8.27 to −3.95; *p* < 0.001). Similarly, participants with uncertain recurrence status reported fewer unmet needs than those with a history of recurrence (B = −4.40, 95% CI −7.37 to −1.43; *p* = 0.004). Language version was independently associated with unmet needs. Participants completing the Russian language version reported significantly fewer unmet needs than those completing the Kazakh language version (B = −3.46, 95% CI −5.10 to −1.82; *p* < 0.001).

Educational attainment and cancer stage were not independently associated with unmet supportive care needs after adjustment for demographic and clinical characteristics.

This pattern was broadly consistent when the model was estimated separately by language (Table 6b,c), with age, sex, and residence remaining significant in both subgroups and the association with confirmed absence of recurrence remaining significant relative to a history of recurrence. Vocational (vs. secondary) education showed a positive association with unmet-needs count, approaching significance in the Russian subsample (β = 0.161, *p* = 0.060) but not in the Kazakh subsample (β = 0.002, *p* = 0.976); given the modest subsample sizes, this difference should be interpreted cautiously.

## 4. Discussion

This study provides the first comprehensive psychometric evaluation of CaSUN in both Russian and Kazakh and, to our knowledge, the first validated survivorship needs assessment measure available for use in Kazakhstan. Beyond establishing psychometric adequacy in each language separately, this study is, to our knowledge, the first to formally test cross-language measurement invariance for the CaSUN, extending its international validation history—which includes Korean, Mexican, and Chinese adaptations—to a genuinely bilingual measurement context. The resulting evidence of metric, and provisionally scalar, equivalence supports meaningful comparison of scores across the Russian and Kazakh versions and has direct relevance for equitable survivorship research and care in multilingual settings. The findings have two complementary implications. First, both language versions showed high internal consistency and evidence supporting a broadly comparable five-factor structure. Second, the pattern of responses identified substantial unmet needs directly relevant to healthcare delivery, particularly access to medical care, current and understandable information, involvement in health management, and coordination between healthcare professionals. Together, these findings support the potential use of CaSUN, not only as a research measure, but also as a structured approach to identifying priorities for patient-centered survivorship services.

Reliability was exceptionally high, with total-scale Cronbach’s alpha scores of 0.966 (Russian) and 0.978 (Kazakh), exceeding the original Australian CaSUN (α = 0.96) [13] and comparable to or exceeding the Korean (α = 0.96) [16] and Mexican (α = 0.95) [18] adaptations. Domain-specific alphas ranged from 0.905 to 0.959, with every domain in both versions exceeding the conventional 0.90 threshold, a narrower and more consistently high range than in several other adaptations, where shorter subscales (particularly Information and Relationships) have shown more variable performance. For comparison, the Korean adaptation’s weakest domain was α = 0.68 [16].

Exploratory factor analysis identified a five-factor solution explaining 68.1% of the variance in the Russian version and 64.2% in the Kazakh version, exceeding the 54% reported in the original validation [13]. The factor structure was broadly consistent with the conceptual organization of CaSUN, while also suggesting some context-specific variation in the clustering of practical and financial concerns. Similar structural variation has been reported in other cultural adaptations. In the Korean CaSUN-K, a distinct ‘financial issues’ factor emerged [16], while the Mexican CaSUN-Mx identified a separate grouping of practical needs [18]. Taken together, these findings suggest that practical and financial survivorship needs may be particularly sensitive to healthcare system and sociocultural context and warrant further investigation in future cross-cultural validation studies.

Unmet needs relating to comprehensive medical care and information provision were highly prevalent: more than half of participants reported unmet needs for the best possible medical care (55.2%), up-to-date information (55.0%), managing health with the healthcare team (53.5%), and access to local services (53.0%) (Figure 1). This mirrors the Korean CaSUN-K, where information-related needs were also the most prevalent domain [16], and the broader literature, in which informational and health system needs are commonly the most frequently endorsed, even though existential and psychosocial needs are typically rated more severe [23]. Psychological concerns were also prominent, with fear of recurrence (49.0%) and emotional support (48.8%) among the ten most frequently endorsed needs (Table 5, Figure 1), underscoring the need for integrated psychosocial support in Kazakhstani survivorship care.

Multivariable regression identified several independent predictors of unmet needs. Younger age was associated with greater unmet needs (B = −0.221, *p* < 0.001), consistent with international evidence linking this to disrupted employment, fertility concerns, and family/financial responsibilities among younger survivors [24]. Male participants reported higher unmet needs than female participants, as reflected by the negative coefficient for female versus male sex (B = −5.08, *p* < 0.001)—a less commonly reported direction, possibly reflecting cultural norms around help—seeking support in Kazakhstan [19,20,25], where women may draw more on informal support networks, or differences in cancer-type distribution within our sample. A history of cancer recurrence was one of the strongest predictors, with participants without a history of recurrence reporting fewer unmet needs (B = −6.11, *p* < 0.001), consistent with evidence that recurrence intensifies psychological distress and treatment burden [26].

An unexpected finding was that rural residents reported fewer unmet needs than urban residents (B = −7.89, *p* < 0.001), contrary to the literature typically linking rural residence with greater unmet needs due to poorer service access [27,28]. Possible explanations include lower expectations of available services among rural survivors, stronger informal family/social support networks substituting for formal care [29], and more detailed elaboration of expectations among urban survivors with greater exposure to specialist services and information.

Kazakh language respondents reported significantly more unmet needs than Russian language respondents (B = −3.46 for Russian vs. Kazakh, *p* < 0.001), independent of residence and other measured covariates. This residual association may reflect unmeasured differences in access to Kazakh language health information, regional healthcare infrastructure, or cultural differences in symptom reporting, highlighting the importance of equally available, culturally adapted survivorship materials in both languages. This pattern—evident already at the item level, where the Kazakh language group having higher prevalence on every one of the twenty-two items showing a significant between-group difference (Table 5)—is also consistent with documented urban–rural disparities in access to cancer-care facilities in Kazakhstan [30], and with cross-cultural differences in unmet-needs-reporting observed in other settings [31]. Given the breadth and consistency of this pattern, survivorship services may wish to pay particular attention to the needs of Kazakh-language-preferring patients when allocating supportive-care resources, pending further research into its specific drivers; we note, however, that the language groups also differed on several sociodemographic and clinical characteristics (Table 1), and that residual confounding from this heterogeneity cannot be fully excluded (see Limitations). Cancer stage and educational attainment were not independently associated with unmet needs after adjustment, consistent with prior survivorship research.

Confirmatory factor analysis provided partial rather than definitive support for the prespecified five-factor model. Incremental fit was good in both language groups (Russian: CFI = 0.975, TLI = 0.973; Kazakh: CFI = 0.973, TLI = 0.971), while RMSEA and SRMR remained above conventional thresholds in both groups (Russian: RMSEA = 0.096, SRMR = 0.116; Kazakh: RMSEA = 0.102, SRMR = 0.099), indicating residual model misfit [32]. The structural findings should therefore be interpreted as supportive but not definitive. RMSEA improved under the binary recoding described above (Section 3.2), but we interpret this improvement with caution: RMSEA is known to be systematically more lenient for coarser categorical data, such that this pattern should not, on its own, be read as evidence that the four-category model is deficient [33]. One contributor may be local dependence between closely related items, particularly items 16 and 17 (see Limitations). Both items concern practical and financial arrangements prompted by a cancer diagnosis (life/travel insurance and legal services, respectively) and had closely matched, comparatively low unmet-need prevalence (20.5% and 19.8%; Table 5), providing a substantive basis for a shared residual source beyond the Quality of Life factor itself. A sensitivity analysis allowing a residual covariance between items 16 and 17 on this basis resolved the non-positive-definite covariance matrix without materially changing model fit (CFI = 0.975, RMSEA = 0.096, SRMR = 0.114 vs. 0.116 at baseline for the Russian sample); an alternative model removing item 17 also resolved this but at greater cost to fit and was not preferred. This pattern, together with cross-loadings on seven to eight items per language group and the divergence in parallel-analysis-suggested factor count (Kazakh: 3 vs. Russian: 5; Supplementary Table S1), suggests the misfit reflects both a few strong local item dependencies and broader structural imprecision, particularly in the Kazakh language version. Measurement invariance testing lends qualified support to this comparability: metric equivalence between the Russian and Kazakh versions was supported, and scalar equivalence was supported by conventional fit index criteria, although the latter could not be corroborated by a formal significance test and should accordingly be treated as provisional pending replication. Future studies in independent samples should examine whether the same factor configuration and local item dependencies are replicated.

These findings have direct implications for survivorship care and healthcare service development in Kazakhstan. More than half of participants reported unmet needs related to medical care, information, participation in health management, or access to local services, suggesting that survivorship needs extend beyond symptom management to the organization and delivery of care. Routine or targeted CaSUN assessment could help oncology services identify patients requiring additional information, psychosocial support, care coordination, or practical assistance and could provide patient-reported data for evaluating service gaps. In particular, routine administration at key transition points—such as completion of primary treatment or entry into survivorship follow-up—could support systematic psychosocial assessment and inform patient-centered care planning across both language groups. The associations with younger age, recurrence, male sex, urban residence, and Kazakh language completion may help inform targeted assessment, although these cross-sectional associations should not be interpreted as causal or used to restrict access to support. Findings from this and other cultural adaptations also suggest that practical and financial concerns warrant explicit attention within survivorship pathways [34,35,36,37].

### Strengths and Limitations

This study has several important strengths. To our knowledge, it is the first study to provide a comprehensive cross-cultural adaptation and psychometric evaluation of CaSUN in both Russian and Kazakh languages. The inclusion of two language versions allowed assessment of the instrument across linguistically diverse populations and provided evidence of measurement equivalence. The study was conducted in a relatively large sample of cancer survivors (*n* = 404), exceeding commonly recommended sample sizes for psychometric validation studies. In addition to evaluating reliability and construct validity, the study explored patterns of unmet supportive care needs and identified demographic and clinical factors associated with these needs, thereby extending the practical relevance of the findings.

Several limitations should also be acknowledged. First, the cross-sectional design does not allow assessment of temporal changes in supportive care needs or causal relationships between patient characteristics and unmet needs. Second, participants were recruited using a convenience sampling approach, which may limit the generalizability of findings to all cancer survivors in Kazakhstan. Third, all information was obtained through self-report questionnaires and may therefore be subject to recall bias and social desirability bias. Fourth, although construct validity was extensively evaluated, test–retest reliability and responsiveness to change were not examined and should be assessed in future longitudinal studies. Finally, the study included survivors with different cancer types and treatment histories, and residual heterogeneity between clinical groups cannot be excluded.

Consistent with STROBE reporting guidance for observational studies [38], we further note that of 457 eligible patients, 53 declined participation; because these individuals could not be characterized, the possibility of non-response bias cannot be ruled out. The single-center setting in Aktobe compounds the generalizability concern raised above: the sample may not fully represent cancer survivors treated in other regions, rural settings, community facilities, or those not currently engaged with oncology services. Finally, cancer type, time since treatment completion, socioeconomic status, comorbidity burden, and access to healthcare services were not captured in the regression model; residual confounding from these unmeasured factors remains possible. Relatedly, because the Russian and Kazakh language groups differed significantly in education, marital status, employment, diagnosis, stage, and recurrence status (Table 1), the independent association of language with unmet-needs count reported in Table 6a should be interpreted with this systematic between-group heterogeneity in mind, notwithstanding statistical adjustment for the measured covariates. Finally, we note as a limitation that parallel analysis supported the prespecified five-factor structure in the Russian sample but suggested only three factors in the Kazakh sample (Supplementary Table S1), indicating that the five-factor solution should be regarded as less stable, and less firmly established, for the Kazakh language version specifically.

The study did not evaluate criterion validity because no validated survivorship needs instrument was available in Russian or Kazakh for comparison. In addition, CFA revealed local dependence involving items 16 (life/travel insurance) and 17 (legal services), which had standardized loadings of 0.99 and 0.97 and an inter-item correlation approaching 1.0; this contributed to a non-positive-definite parameter covariance matrix. Although the model converged and the loadings were interpretable, this finding, together with the elevated RMSEA and SRMR, warrants caution when interpreting structural validity. A sensitivity analysis introducing a theoretically motivated residual covariance between these items (detailed in Section 4) resolved the non-positive-definite matrix without materially altering overall model fit, and was preferred to item deletion, which exacted a greater cost in fit. Replication in an independent sample is needed to determine whether these items consistently capture overlapping content and whether theoretically justified model refinement or item revision is appropriate.

## 5. Conclusions

This study provides initial evidence supporting the reliability, construct validity, and cross-language comparability of the Russian and Kazakh versions of CaSUN among cancer survivors in Kazakhstan. To our knowledge, this is the first study to translate, culturally adapt, and psychometrically validate CaSUN in Russian and Kazakh, and the first to formally evaluate cross-language measurement invariance for this instrument, thereby extending its international validation history to Kazakhstan’s bilingual healthcare context. Both versions demonstrated excellent internal consistency, and factor analyses broadly supported a five-factor organization, although residual CFA misfit indicates that further structural validation is warranted.

In particular, the five-factor structure should be regarded as provisional for the Kazakh language version specifically, given the divergent exploratory factor count observed in that group. We further emphasize that although the residual-covariance model was rendered admissible (free of Heywood cases or non-positive-definite matrices) in both language groups, its absolute fit indices (RMSEA and SRMR) remained above conventional thresholds; the measurement-invariance findings reported here should therefore be regarded as exploratory rather than as resting on a fully established, well-fitting baseline structure, and should be re-examined when such a baseline has been achieved and confirmed in an independent sample.

The findings further revealed a substantial burden of unmet supportive care needs among cancer survivors, particularly in the domains of information provision, access to healthcare services, care coordination, and psychosocial support. Younger survivors, individuals with a history of cancer recurrence, male participants, urban residents, and respondents completing the Kazakh language version reported significantly greater unmet needs.

The availability of culturally adapted Russian and Kazakh CaSUN versions provides healthcare professionals, researchers, and policymakers with a practical approach to systematically assessing survivorship needs in a multilingual healthcare setting. Integration of needs assessment into survivorship pathways could support earlier identification of patients requiring additional information, psychosocial support, care coordination, or practical assistance and could inform patient-centered service development. Future longitudinal and multicentre studies should examine test–retest reliability, responsiveness, structural stability, and changes in supportive care needs across the survivorship trajectory.

## Figures and Tables

**Table 1:** Sociodemographic and clinical characteristics of participants by language group (*n* = 404).

Characteristic	Russian (*n* = 201)	Kazakh (*n* = 203)	Total (*n* = 404)	*p*
**Age, years (mean ± SD)**	60.6 ± 9.5	60.7 ± 10.1	60.7 ± 9.8	0.921
**Sex**				0.969
Male	63 (31.3%)	65 (32.0%)	128 (31.7%)	
Female	138 (68.7%)	138 (68.0%)	276 (68.3%)	
**Residence**				0.696
Urban	138 (68.7%)	144 (70.9%)	282 (69.8%)	
Rural	63 (31.3%)	59 (29.1%)	122 (30.2%)	
**Education**				**0.020**
Secondary	35 (17.4%)	61 (30.0%)	96 (23.8%)	
Vocational	108 (53.7%)	90 (44.3%)	198 (49.0%)	
Higher	57 (28.4%)	52 (25.6%)	109 (27.0%)	
Other	1 (0.5%)	0 (0.0%)	1 (0.2%)	
**Marital status**				**<0.001**
Married	88 (43.8%)	114 (56.2%)	202 (50.0%)	
Not married	24 (11.9%)	16 (7.9%)	40 (9.9%)	
Has children	47 (23.4%)	18 (8.9%)	65 (16.1%)	
Not married, has children	11 (5.5%)	9 (4.4%)	20 (5.0%)	
Married, has children	31 (15.4%)	46 (22.7%)	77 (19.1%)	
**Employment status**				**0.004**
Employed, full-time	34 (16.9%)	39 (19.2%)	73 (18.1%)	
Employed, part-time	34 (16.9%)	34 (16.7%)	68 (16.8%)	
Temporarily unemployed	46 (22.9%)	20 (9.9%)	66 (16.3%)	
Retired	87 (43.3%)	110 (54.2%)	197 (48.8%)	
**Cancer diagnosis**				**<0.001**
Endometrial	18 (9.0%)	9 (4.4%)	27 (6.7%)	
Cervical	18 (9.0%)	24 (11.8%)	42 (10.4%)	
Breast	48 (23.9%)	59 (29.1%)	107 (26.5%)	
Ovarian	8 (4.0%)	6 (3.0%)	14 (3.5%)	
Lung	5 (2.5%)	20 (9.9%)	25 (6.2%)	
Gastric	20 (10.0%)	9 (4.4%)	29 (7.2%)	
Nasal	5 (2.5%)	2 (1.0%)	7 (1.7%)	
Prostate	9 (4.5%)	1 (0.5%)	10 (2.5%)	
Hematologic (blood)	8 (4.0%)	18 (8.9%)	26 (6.4%)	
Kidney	62 (30.8%)	55 (27.1%)	117 (29.0%)	
**Disease stage**				**0.005**
I	33 (16.4%)	38 (18.7%)	71 (17.6%)	
II	121 (60.2%)	111 (54.7%)	232 (57.4%)	
III	29 (14.4%)	15 (7.4%)	44 (10.9%)	
IV	6 (3.0%)	6 (3.0%)	12 (3.0%)	
Unknown	12 (6.0%)	33 (16.3%)	45 (11.1%)	
**Recurrence**				**0.026**
Yes	31 (15.4%)	49 (24.1%)	80 (19.8%)	
No	145 (72.1%)	121 (59.6%)	266 (65.8%)	
Unknown	25 (12.4%)	33 (16.3%)	58 (14.4%)	

Bold *p*-values indicate statistically significant differences between language groups (*p* < 0.05).

**Table 2:** Reliability and construct validity of the Russian and Kazakh versions of CaSUN.

**A. Internal Consistency Reliability**			
**Domain**	**Number of Items**	**Russian Version Cronbach’s α**	**Kazakh Version Cronbach’s α**
Existential Survivorship	7	0.948	0.959
Comprehensive Cancer Care	6	0.930	0.931
Information	3	0.909	0.909
Quality of Life	9	0.905	0.930
Relationships	10	0.915	0.956
**Total scale**	**35**	**0.966**	**0.978**
**B. Construct Validity and Exploratory Factor Analysis**			
**Psychometric Property**	**Russian Version**		**Kazakh Version**
Sample size	201		203
Kaiser–Meyer–Olkin (KMO)	0.920		0.940
Bartlett’s χ^2^	7349.95		9081.48
Degrees of freedom	595		595
*p*-value	<0.001		<0.001
Number of extracted factors	5		5
Total variance explained (%)	68.1		64.2

Bold values indicate the total-scale reliability estimates (number of items and Cronbach’s α).

**Table 3:** (**a**) Confirmatory factor analysis fit indices by language group (WLSMV estimator, ordinal/polychoric). (**b**) Inter-factor correlations by language group (WLSMV estimator).

**(a)**									
**Language**	**Model**	***n***	**χ^2^ (Scaled)**	**df**	**CFI**	**TLI**	**RMSEA**	**SRMR**	**Heywood Case?**
Russian	Baseline 5-factor CFA (no residual covariance)	201	1570.996	550	0.975	0.973	0.096	0.116	Yes—item16 (est.std = −0.001)
Russian	Sensitivity: item 16 ↔ item 17 residual covariance	201	1556.687	549	0.975	0.973	0.096	0.114	No
Russian	Sensitivity: item 17 removed	201	1503.356	517	0.961	0.958	0.098	0.110	No
Kazakh	Baseline 5-factor CFA (no residual covariance)	203	1708.544	550	0.973	0.971	0.102	0.099	No (borderline non-positive-definite vcov)
Kazakh	Sensitivity: item 16 ↔ item 17 residual covariance	203	1684.221	549	0.974	0.972	0.101	0.098	No
Kazakh	Sensitivity: item 17 removed	203	1601.448	517	0.973	0.970	0.102	0.095	No
(**b**)									
**Factor 1**		**Factor 2**				**Russian r**			**Kazakh r**
Information		Comprehensive Care				0.856			0.918
Information		Quality of Life				0.597			0.702
Information		Relationships				0.782			0.830
Information		Existential Survivorship				0.812			0.850
Comprehensive Care		Quality of Life				0.424			0.583
Comprehensive Care		Relationships				0.678			0.783
Comprehensive Care		Existential Survivorship				0.728			0.835
Quality of Life		Relationships				0.621			0.787
Quality of Life		Existential Survivorship				0.626			0.735
Relationships		Existential Survivorship				0.801			0.902

All models estimated in R (lavaan), WLSMV estimator, items treated as ordered categorical (0–3). All inter-factor correlations are large (r > 0.40) in both language groups. Correlations are consistently higher in the Kazakh sample across factor pairs, consistent with the broader item-level irregularities (cross-loadings, elevated loadings, and the divergent parallel analysis factor count) reported in Supplementary Table S1 and discussed in the Strengths and Limitations section.

**Table 4:** Measurement invariance across Russian and Kazakh language versions.

**Model**	**CFI**	**TLI**	**RMSEA**	**SRMR**	**ΔCFI**	**ΔRMSEA**	**ΔSRMR**
Configural	0.974	0.972	0.099	0.107	-	-	-
Metric	0.977	0.976	0.092	0.111	+0.003	−0.007	+0.004
Scalar	0.974	0.973	0.096	0.107	−0.003	+0.004	−0.004

**Table 5:** Prevalence of unmet needs by CaSUN item and language group, ranked by overall prevalence.

CaSUN Item	Russian (*n* = 201)	Kazakh (*n* = 203)	Total (*n* = 404)	*p*
4. Best medical care	107 (53.2%)	116 (57.1%)	223 (55.2%)	0.490
1. Up-to-date information	100 (49.8%)	122 (60.1%)	222 (55.0%)	**0.047**
6. Manage health with team	102 (50.7%)	114 (56.2%)	216 (53.5%)	0.322
5. Local health care services	105 (52.2%)	109 (53.7%)	214 (53.0%)	0.847
3. Understandable information	104 (51.7%)	105 (51.7%)	209 (51.7%)	1.000
2. Information for family/partner	102 (50.7%)	106 (52.2%)	208 (51.5%)	0.844
7. Doctors talk to each other	98 (48.8%)	106 (52.2%)	204 (50.5%)	0.551
19. Recurrence concerns	80 (39.8%)	118 (58.1%)	198 (49.0%)	**<0.001**
10. Reduce stress in my life	79 (39.3%)	118 (58.1%)	197 (48.8%)	**<0.001**
20. Emotional support for me	82 (40.8%)	115 (56.7%)	197 (48.8%)	**0.002**
11. Manage side effects	77 (38.3%)	111 (54.7%)	188 (46.5%)	**0.001**
8. Complaints addressed	82 (40.8%)	100 (49.3%)	182 (45.0%)	0.107
30. Changes to beliefs	75 (37.3%)	101 (49.8%)	176 (43.6%)	**0.015**
9. Complementary/alternative therapy	78 (38.8%)	96 (47.3%)	174 (43.1%)	0.105
29. Move on with my life	74 (36.8%)	97 (47.8%)	171 (42.3%)	**0.033**
32. Survivor expectations	73 (36.3%)	90 (44.3%)	163 (40.3%)	0.123
31. Acknowledging the impact	72 (35.8%)	87 (42.9%)	159 (39.4%)	0.178
21. Support partner/family	63 (31.3%)	95 (46.8%)	158 (39.1%)	**0.002**
35. Make my life count	62 (30.8%)	96 (47.3%)	158 (39.1%)	**0.001**
34. Spiritual beliefs	64 (31.8%)	87 (42.9%)	151 (37.4%)	**0.029**
33. Decisions about my life	68 (33.8%)	83 (40.9%)	151 (37.4%)	0.173
24. Talk to others	54 (26.9%)	95 (46.8%)	149 (36.9%)	**<0.001**
12. Changes to quality of life	58 (28.9%)	85 (41.9%)	143 (35.4%)	**0.009**
22. Impact on my relationship	46 (22.9%)	93 (45.8%)	139 (34.4%)	**<0.001**
23. New relationships	42 (20.9%)	87 (42.9%)	129 (31.9%)	**<0.001**
26. Changes to my body	48 (23.9%)	68 (33.5%)	116 (28.7%)	**0.043**
14. Employment/keeping job	47 (23.4%)	65 (32.0%)	112 (27.7%)	0.068
25. Handle social/work situations	45 (22.4%)	67 (33.0%)	112 (27.7%)	**0.023**
18. Accessible hospital parking	39 (19.4%)	66 (32.5%)	105 (26.0%)	**0.004**
28. Ongoing case manager	31 (15.4%)	65 (32.0%)	96 (23.8%)	**<0.001**
15. Financial support	31 (15.4%)	64 (31.5%)	95 (23.5%)	**<0.001**
16. Life/travel insurance	29 (14.4%)	54 (26.6%)	83 (20.5%)	**0.004**
17. Legal services	29 (14.4%)	51 (25.1%)	80 (19.8%)	**0.010**
27. Problems with sex life	17 (8.5%)	48 (23.6%)	65 (16.1%)	**<0.001**
13. Fertility problems	28 (13.9%)	34 (16.7%)	62 (15.3%)	0.517

Values are *n* (%) endorsing an unmet need (response 2 or 3) out of the group total. *p*-values from Pearson’s chi-squared test comparing Russian vs. Kazakh language groups; bold indicates *p* < 0.05.

**Table 6:** (**a**) Multivariable regression analysis of factors associated with the number of unmet supportive care needs: total sample (*n* = 403). (**b**) Multivariable regression analysis: Russian language subsample (*n* = 200). (**c**) Multivariable regression analysis: Kazakh language subsample (*n* = 203).

(**a**)				
**Predictor**	**B**	**95% CI**	**β**	***p***
Age	−0.221	−0.305, −0.137	−0.212	**<0.001**
Sex: Female vs. Male	−5.084	−6.815, −3.353	−0.233	**<0.001**
Residence: Rural vs. Urban	−7.887	−9.762, −6.011	−0.356	**<0.001**
Education: Vocational vs. Secondary	1.464	−0.600, 3.527	0.072	0.164
Education: Higher vs. Secondary	−0.105	−2.355, 2.146	−0.005	0.927
Stage: II vs. I	0.715	−1.585, 3.015	0.035	0.541
Stage: III vs. I	−1.386	−4.603, 1.831	−0.042	0.397
Stage: IV vs. I	−2.562	−7.689, 2.565	−0.043	0.327
Stage: Unknown vs. I	−2.226	−5.549, 1.097	−0.069	0.189
Recurrence: No vs. Yes	−6.113	−8.273, −3.954	−0.285	**<0.001**
Recurrence: Unknown vs. Yes	−4.399	−7.369, −1.428	−0.152	**0.004**
Language: Russian vs. Kazakh	−3.460	−5.095, −1.825	−0.170	**<0.001**
(**b**)				
**Predictor**	**B**	**95% CI**	**β**	***p***
Age	−0.185	−0.297, −0.074	−0.201	**0.001**
Sex: Female vs. Male	−4.722	−6.931, −2.513	−0.252	**<0.001**
Residence: Rural vs. Urban	−5.821	−8.152, −3.490	−0.311	**<0.001**
Education: Vocational vs. Secondary	2.802	−0.124, 5.728	0.161	0.060
Education: Higher vs. Secondary	−0.632	−3.810, 2.547	−0.033	0.695
Stage: II vs. I	2.240	−0.748, 5.229	0.126	0.141
Stage: III vs. I	1.595	−2.250, 5.439	0.064	0.414
Stage: IV vs. I	−4.249	−10.825, 2.326	−0.084	0.204
Stage: Unknown vs. I	−1.820	−7.336, 3.696	−0.050	0.516
Recurrence: No vs. Yes	−4.877	−7.805, −1.949	−0.253	**0.001**
Recurrence: Unknown vs. Yes	−3.961	−8.187, 0.266	−0.151	0.066
(**c**)				
**Predictor**	**B**	**95% CI**	**β**	***p***
Age	−0.249	−0.377, −0.121	−0.225	**<0.001**
Sex: Female vs. Male	−5.051	−7.770, −2.332	−0.212	**0.001**
Residence: Rural vs. Urban	−9.473	−12.492, −6.455	−0.387	**<0.001**
Education: Vocational vs. Secondary	0.047	−2.998, 3.092	0.002	0.976
Education: Higher vs. Secondary	−0.011	−3.288, 3.266	0.000	0.995
Stage: II vs. I	−0.920	−4.465, 2.624	−0.041	0.609
Stage: III vs. I	−5.164	−10.742, 0.414	−0.121	0.069
Stage: IV vs. I	−0.871	−8.948, 7.206	−0.013	0.832
Stage: Unknown vs. I	−2.244	−6.751, 2.263	−0.074	0.327
Recurrence: No vs. Yes	−7.445	−10.711, −4.178	−0.328	**<0.001**
Recurrence: Unknown vs. Yes	−4.382	−8.656, −0.109	−0.145	**0.045**

Reference categories: male sex, urban residence, secondary education, stage I disease, recurrence present, and Kazakh language version. One participant with unclassified/’other’ education (*n* = 1) was excluded due to insufficient category size. R^2^ = 0.407, Adjusted R^2^ = 0.387. β = standardized coefficient. One participant with unclassified/’other’ education was excluded (*n* = 200 of 201). R^2^ = 0.367, Adjusted R^2^ = 0.330. Reference categories as in Table 6a. R^2^ = 0.431, Adjusted R^2^ = 0.399. Reference categories as in Table 6a. No respondent in the Kazakh subsample reported ‘other’ education; bold indicates *p* < 0.05.

## Data Availability

The data presented in this study are available from the corresponding author upon reasonable request. The data are not publicly available because they contain participant-level clinical and sociodemographic information.

## Supplementary Materials

The following supporting information can be downloaded at https://www.mdpi.com/article/10.3390/healthcare14183012/s1, Table S1: Exploratory factor analysis (EFA) summary by language group (polychoric correlations, principal-axis factoring, promax rotation). Table S2: Russian sample-EFA loadings, communalities, cross-loadings, and factor correlations (*n* = 201). Table S3: Kazakh sample-EFA loadings, communalities, cross-loadings, and factor correlations (*n* = 203). Table S4: Confirmatory factor analysis (CFA): standardized loadings and residual variances by language group (WLSMV estimator, prespecified five-factor model). Table S5: CFA model convergence and warnings by language group (baseline model, no residual covariance). Table S6: Category frequencies for all 35 CaSUN items, by language group.

## Abbreviations

CaSUN—Cancer Survivors’ Unmet Needs Measure; CFA—confirmatory factor analysis; CFI—Comparative Fit Index; CI—confidence interval; EFA—exploratory factor analysis; IQR—interquartile range; KMO—Kaiser–Meyer–Olkin (measure of sampling adequacy); RMSEA—Root Mean Square Error of Approximation; SD—standard deviation; SRMR—Standardized Root Mean Square Residual; TLI—Tucker–Lewis Index.

## References

[1] American Association for Cancer Research (2025). AACR Cancer Progress Report 2025.

[2] Sung H., Ferlay J., Siegel R.L., Laversanne M., Soerjomataram I., Jemal A., Bray F. (2021). Global Cancer Statistics 2020: GLOBOCAN Estimates of Incidence and Mortality Worldwide for 36 Cancers in 185 Countries. CA Cancer J. Clin..

[3] Andreu Y., Gil-Juliá B., Picazo C., García-Conde A., Soto-Rubio A. (2025). Unmet Supportive Care Needs in Cancer Survivors in Spain: A Multicentre Cross-Sectional Study on Prevalence and Sociodemographic and Disease-Related Risk Factors. Curr. Oncol..

[4] Maheu C., Tock W.L., Fisher P., Galica J., Singh M., Centeno I., Hébert M., Moran C., Pietruczuk P., Dinkel A. (2025). Systematic Review of Fear of Cancer Recurrence Patient-Reported Outcome Measures: Evaluating Methodological Quality and Measurement Properties Using the COSMIN Checklist. Healthcare.

[5] Yang T., Zhu Z., Shi J., Tong L., Yang J., Mei S., Ren H. (2025). Association among Financial Toxicity, Depression and Fear of Cancer Recurrence in Young Breast Cancer Patient-Family Caregiver Dyads: An Actor-Partner Interdependence Mediation Model. BMC Psychiatry.

[6] Chen M., Li R., Chen Y., Ding G., Song J., Hu X., Jin C. (2022). Unmet Supportive Care Needs and Associated Factors: Evidence from 4195 Cancer Survivors in Shanghai, China. Front. Oncol..

[7] Herbert S.-L., Payerl A.S., Prange M., Löb S., Büchel J., Scherer-Quenzer A., Kiesel M., Wöckel A., Faller H., Meng K. (2025). Supportive Care and Information Needs in Relation to Quality of Life among Patients with Breast Cancer and Gynaecological Cancer during the Time of Treatment. Arch. Gynecol. Obstet..

[8] Miranda K.W., Musher B.L., Badr H.J. (2025). Unmet Supportive Care Needs in Patients with Advanced Cancer and Its Impact on Distress. BMC Palliat. Care.

[9] Wang B., Hu X., Ding W., Guo H., Ren X. (2025). Supportive Care Needs and Quality of Life among Cancer Patients in China: A Cross-Sectional Study. PLoS ONE.

[10] Weaver R., O’Connor M., Sobhi S., Carey Smith R., Halkett G. (2020). The Unmet Needs of Patients with Sarcoma. Psychooncology.

[11] Thiessen M., Harris D., Tang P.A., Raffin Bouchal S., Sinclair S. (2024). Examining the Development of Information Needs Assessment Tools for Use in the Cancer Context: A Scoping and Critical Review. Palliat. Support. Care.

[12] Richardson A., Medina J., Brown V., Sitzia J. (2007). Patients’ Needs Assessment in Cancer Care: A Review of Assessment Tools. Support. Care Cancer.

[13] Hodgkinson K., Butow P., Hunt G.E., Pendlebury S., Hobbs K.M., Wain G. (2007). The Development and Evaluation of a Measure to Assess Cancer Survivors’ Unmet Supportive Care Needs: The CaSUN (Cancer Survivors’ Unmet Needs Measure). Psycho-Oncology.

[14] Fang S.-Y., Cheng H.-R., Lin C.-Y. (2018). Validation of the Modified Chinese Cancer Survivor’s Unmet Needs (CaSUN-C) for Women with Breast Cancer. Psycho-Oncology.

[15] Li Q., Xu Y., Lin Y., Li J., Huang W., Chen Y. (2020). Psychometric Properties of the Chinese Version of the Cancer Survivors’ Unmet Needs Measure. Eur. J. Oncol. Nurs..

[16] Kang D., Lee J., Kim S., Nam H., Kong S., Shim S., Lee J.K., Jung W., Shin S., Kim H.K. (2023). Psychometric Validation of the Korean Version of the Cancer Survivors’ Unmet Needs (CaSUN) Scale among Korean Non–Small Cell Lung Cancer Survivors. Cancer Res. Treat..

[17] Xu X., Liang X., Yin S. (2024). Validity and Reliability of a Simplified Chinese Version of Cancer Survivors’ Unmet Needs Scale (CaSUN). Psycho-Oncology.

[18] Mazatán Orozco R., Torres Alegre C., Zayas-Villanueva O.A., Reynoso González O.U., Fraga-Sastrias J.-M., Sierra-Murguía M.A. (2024). Validation and Adaptation of the Spanish Version of Cancer Survivors’ Unmet Needs Survey (CaSUN-MX) for Use in the Mexican Adult Oncology Population. Salud Ment..

[19] Thibault H., Caron J.-F., Thibault H., Caron J.-F. (2022). An Overview of Shame and Its Manifestation in Central Asia. Uyat and the Culture of Shame in Central Asia.

[20] Akhmetova S., Durrani N., Karabay A. (2025). Intimate Partner Violence and the Culture of Shame in Kazakhstan: Voices of Survivors and Violence Against Women Professionals. Violence Against Women.

[21] Beaton D.E., Bombardier C., Guillemin F., Ferraz M.B. (2000). Guidelines for the Process of Cross-Cultural Adaptation of Self-Report Measures. Spine.

[22] Wild D., Grove A., Martin M., Eremenco S., McElroy S., Verjee-Lorenz A., Erikson P. (2005). Principles of Good Practice for the Translation and Cultural Adaptation Process for Patient-Reported Outcomes (PRO) Measures: Report of the ISPOR Task Force for Translation and Cultural Adaptation. Value Health.

[23] Harrison J.D., Young J.M., Price M.A., Butow P.N., Solomon M.J. (2009). What Are the Unmet Supportive Care Needs of People with Cancer? A Systematic Review. Support. Care Cancer.

[24] Altherr A., Bolliger C., Kaufmann M., Dyntar D., Scheinemann K., Michel G. (2023). Education, Employment, and Financial Outcomes in Adolescent and Young Adult Cancer Survivors: A Systematic Review. Curr. Oncol..

[25] Fish J.A., Prichard I., Ettridge K., Grunfeld E.A., Wilson C. (2015). Psychosocial Factors That Influence Men’s Help-Seeking for Cancer Symptoms: A Systematic Synthesis of Mixed Methods Research. Psychooncology.

[26] Kim M.-L., Kim Y., Choe Y.-R. (2025). Unmet Needs Mediate the Impact of Fear of Cancer Recurrence on Screening Participation Among Cancer Survivors: A Cross-Sectional Study. Healthcare.

[27] Sepassi A., Li M., Zell J.A., Chan A., Saunders I.M., Mukamel D.B. (2024). Rural-Urban Disparities in Colorectal Cancer Screening, Diagnosis, Treatment, and Survivorship Care: A Systematic Review and Meta-Analysis. Oncologist.

[28] Stout N.L., Boatman D., Rice M., Branham E., Miller M., Salyer R. (2024). Unmet Needs and Care Delivery Gaps Among Rural Cancer Survivors. J. Patient Exp..

[29] Butow P.N., Phillips F., Schweder J., White K., Underhill C., Goldstein D. (2012). Psychosocial Well-Being and Supportive Care Needs of Cancer Patients Living in Urban and Rural/Regional Areas: A Systematic Review. Support. Care Cancer.

[30] Akhmedullin R., Aimyshev T., Zhakhina G., Yerdessov S., Beyembetova A., Ablayeva A., Biniyazova A., Seyil T., Abdukhakimova D., Segizbayeva A. (2024). In-Depth Analysis and Trends of Cancer Mortality in Kazakhstan: A Joinpoint Analysis of Nationwide Healthcare Data 2014–2022. BMC Cancer.

[31] Lam W.W.T., Au A.H.Y., Wong J.H.F., Lehmann C., Koch U., Fielding R., Mehnert A. (2011). Unmet Supportive Care Needs: A Cross-Cultural Comparison Between Hong Kong Chinese and German Caucasian Women with Breast Cancer. Breast Cancer Res. Treat..

[32] Hu L.-T., Bentler P.M. (1999). Cutoff Criteria for Fit Indexes in Covariance Structure Analysis: Conventional Criteria versus New Alternatives. Struct. Equ. Model..

[33] Shi D., Maydeu-Olivares A., Rosseel Y. (2020). Assessing Fit in Ordinal Factor Analysis Models: SRMR vs. RMSEA. Struct. Equ. Model..

[34] Dorey G., Cabaset S., Richard A., Dehler A., Kudre D., Schneider-Mörsch B., Sperisen N., Schmid M., Rohrmann S. (2021). National Study for Multidisciplinary Outpatient Oncological Rehabilitation: Online Survey to Support Revised Quality and Performance Criteria. Support. Care Cancer.

[35] Reese C., Weis J., Schmucker D., Mittag O. (2017). Development of Practice Guidelines for Psychological Interventions in the Rehabilitation of Patients with Oncological Disease (Breast, Prostate, or Colorectal Cancer): Methods and Results. Psychooncology.

[36] Azzani M., Atroosh W.M., Anbazhagan D., Kumarasamy V., Abdalla M.M.I. (2024). Describing Financial Toxicity among Cancer Patients in Different Income Countries: A Systematic Review and Meta-Analysis. Front. Public Health.

[37] Jin D.L., Kim Y.A., Lee S.J., Seo H.J., Yoon S.J. (2025). Factors Associated with Unmet Supportive Care Needs among Adult Cancer Survivors in South Korea: A Cross-Sectional Survey. J. Cancer Surviv..

[38] von Elm E., Altman D.G., Egger M., Pocock S.J., Gøtzsche P.C., Vandenbroucke J.P., STROBE Initiative (2008). The Strengthening the Reporting of Observational Studies in Epidemiology (STROBE) Statement: Guidelines for Reporting Observational Studies. J. Clin. Epidemiol..

