# Supplementary material for: Cross-Cultural Adaptation and Psychometric Validation of the Cancer Survivors’ Unmet Needs Measure (CaSUN) in Russian- and Kazakh-Speaking Cancer Survivors in Kazakhstan
Source: Healthcare (Basel). 2026 Sep 14;14(18):3012. doi: 10.3390/healthcare14183012 (PMC13606774; doi:10.3390/healthcare14183012)
Supplement: Supplementary file 1 [file healthcare-14-03012-s001.zip › healthcare-4502268-supplementary.pdf]

Supplementary Materials

Cross-Cultural Adaptation and Psychometric Validation of the Cancer Survivors' Unmet Needs Measure (CaSUN) in Russian- and Kazakh-Speaking Cancer Survivors in Kazakhstan

Gulnar Karabasova, Kerbez Kimatova, Nurgul Abenova, Anar Tulyayeva, Zalika Klemenc-Ketiš and Perizat Aitmaganbet

Table S1. Exploratory factor analysis (EFA) summary by language group (polychoric correlations, principal-axis factoring, promax rotation).

Language	N	Parallel analysis: suggested factors	Prespecified factors used	EFA RMSEA	EFA TLI
Russian	201	5	5	0.501	−0.031
Kazakh	203	3	5	0.490	0.050

Parallel analysis (polychoric correlations, 20 resamples) supported the prespecified five-factor structure in the Russian sample but suggested only three factors in the Kazakh sample, indicating potential instability of the five-factor solution in the Kazakh-language group. EFA RMSEA/TLI are internal fit indices from the psych::fa() solution (based on the reduced correlation matrix) and are not directly comparable to the lavaan CFA fit indices reported in Table 3 of the main text.

Table S2. Russian sample - EFA loadings, communalities, cross-loadings, and factor correlations (N = 201).

Item	PA4	PA1	PA3	PA2	PA5	h^2	Cross-loading (≥.30 on >1 factor)
item1	**0.467**	0.106	0.035	-0.073	**0.383**	0.716	**Yes**
item2	**0.502**	0.135	0.110	-0.021	0.195	0.678	
item3	**0.601**	0.250	0.023	0.007	0.104	0.804	
item4	**0.679**	**0.375**	-0.047	0.025	-0.151	0.754	**Yes**
item5	**0.907**	0.119	-0.138	0.173	-0.133	0.800	
item6	**0.990**	-0.138	-0.080	0.081	0.030	0.784	
item7	**0.873**	-0.124	0.112	-0.121	0.098	0.787	
item8	**0.918**	-0.123	0.079	-0.147	0.045	0.771	
item9	**0.915**	-0.053	-0.003	0.019	-0.051	0.724	
item10	0.122	-0.124	0.047	-0.035	**0.914**	0.830	
item11	0.018	-0.050	0.035	-0.045	**0.920**	0.798	
item12	0.019	-0.010	-0.028	0.150	**0.691**	0.591	
item13	-0.148	0.010	-0.172	**0.489**	**0.413**	0.447	**Yes**
item14	-0.095	-0.099	0.060	**0.776**	0.275	0.817	

item15	-0.006	-0.002	0.008	**1.008**	-0.075	0.941	
item16	0.045	-0.004	0.082	**1.035**	-0.211	0.945	
item17	0.086	-0.006	0.057	**1.025**	-0.155	0.976	
item18	0.168	0.005	0.060	**0.799**	0.032	0.834	
item19	**0.311**	0.267	0.191	0.135	0.100	0.689	
item20	0.280	**0.335**	0.098	0.114	0.188	0.731	
item21	0.004	**0.451**	**0.388**	-0.123	0.166	0.729	**Yes**
item22	0.118	-0.172	**0.677**	0.048	0.124	0.551	
item23	0.025	0.001	**0.924**	0.021	-0.168	0.712	
item24	0.159	0.142	**0.845**	-0.051	-0.167	0.855	
item25	0.119	-0.031	**0.872**	0.071	-0.077	0.820	
item26	0.004	-0.058	**0.878**	-0.110	0.111	0.764	
item27	-0.275	0.096	**0.694**	0.157	0.042	0.541	
item28	-0.117	0.058	**0.486**	0.012	**0.340**	0.542	**Yes**
item29	0.047	**0.479**	0.000	0.016	**0.436**	0.810	**Yes**
item30	0.064	**0.486**	-0.012	-0.028	**0.413**	0.750	**Yes**
item31	0.168	**0.606**	-0.072	0.039	0.192	0.742	
item32	0.095	**1.001**	-0.123	-0.005	-0.059	0.897	
item33	-0.028	**1.013**	0.056	-0.024	-0.153	0.828	
item34	-0.004	**0.975**	0.049	-0.032	-0.089	0.858	
item35	0.065	**0.918**	0.058	-0.014	-0.086	0.880	

Factor correlations (Russian):

	PA4	PA1	PA3	PA2	PA5
**PA4**	1.000	0.740	0.597	0.252	0.598
**PA1**	0.740	1.000	0.688	0.455	0.754
**PA3**	0.597	0.688	1.000	0.421	0.665
**PA2**	0.252	0.455	0.421	1.000	0.551
**PA5**	0.598	0.754	0.665	0.551	1.000

Bold loadings/cross-loading flags indicate values ≥ .30 (loadings) as specified. h^2 = communality. Estimator: WLSMV-consistent polychoric correlations, principal-axis factoring, promax rotation.

Table S3. Kazakh sample - EFA loadings, communalities, cross-loadings, and factor correlations (N = 203).

Item	PA1	PA5	PA2	PA3	PA4	h²	Cross-loading (≥.30 on >1 factor)
item1	**0.811**	-0.114	0.183	-0.074	0.160	0.737	
item2	**0.718**	0.078	-0.109	0.074	0.234	0.810	
item3	**0.934**	0.004	0.020	-0.063	0.031	0.849	
item4	**1.010**	-0.227	0.201	0.005	-0.157	0.782	
item5	**0.817**	0.121	0.043	0.009	-0.106	0.816	
item6	**0.915**	-0.032	-0.207	0.014	0.088	0.719	
item7	**0.991**	-0.121	-0.194	0.112	-0.036	0.766	
item8	**0.975**	-0.068	-0.006	0.034	-0.103	0.813	
item9	**0.759**	0.293	-0.189	-0.106	0.085	0.794	
item10	**0.334**	**0.318**	0.226	-0.170	**0.381**	0.845	**Yes**
item11	0.294	**0.368**	-0.003	-0.006	**0.394**	0.790	**Yes**
item12	0.145	**0.389**	-0.015	-0.195	**0.673**	0.826	**Yes**
item13	-0.171	**-0.401**	0.234	**0.366**	**0.781**	0.798	**Yes**
item14	-0.153	0.065	**0.927**	-0.012	0.135	0.943	
item15	-0.149	-0.031	**1.082**	-0.051	0.036	0.975	
item16	-0.082	0.026	**0.971**	-0.003	0.020	0.917	
item17	-0.009	-0.029	**1.078**	-0.069	-0.043	0.972	
item18	0.224	0.065	**0.764**	-0.034	-0.028	0.820	
item19	0.200	**0.496**	0.297	0.092	0.020	0.942	
item20	**0.363**	**0.466**	0.151	0.101	-0.041	0.893	**Yes**
item21	**0.399**	0.297	0.178	0.200	-0.050	0.838	
item22	0.243	0.055	**0.326**	**0.317**	0.060	0.720	**Yes**
item23	0.036	0.067	**0.320**	**0.494**	0.101	0.788	**Yes**
item24	0.077	0.198	-0.053	**0.625**	0.139	0.770	
item25	0.204	0.136	0.115	**0.632**	-0.151	0.823	
item26	0.250	0.133	0.143	**0.473**	-0.019	0.760	
item27	-0.034	-0.103	-0.096	**1.008**	0.127	0.832	

item28	0.032	0.100	0.018	**0.824**	0.010	0.881	
item29	-0.003	**0.779**	0.079	0.077	0.047	0.836	
item30	-0.064	**0.846**	-0.052	0.146	0.109	0.868	
item31	0.197	**0.619**	0.042	0.142	0.010	0.865	
item32	0.279	**0.560**	0.015	0.162	-0.023	0.856	
item33	0.212	**0.656**	-0.107	**0.302**	-0.138	0.892	**Yes**
item34	0.289	**0.641**	-0.069	0.196	-0.171	0.853	
item35	0.280	**0.834**	0.096	-0.216	-0.148	0.841	

Factor correlations (Kazakh):

	**PA1**	**PA5**	**PA2**	**PA3**	**PA4**
**PA1**	1.000	0.807	0.500	0.654	0.429
**PA5**	0.807	1.000	0.639	0.720	0.516
**PA2**	0.500	0.639	1.000	0.693	0.567
**PA3**	0.654	0.720	0.693	1.000	0.461
**PA4**	0.429	0.516	0.461	0.567	1.000

Bold loadings/cross-loading flags indicate values ≥ .30 (loadings) as specified. h² = communality. Estimator: WLSMV-consistent polychoric correlations, principal-axis factoring, promax rotation.

Table S4. Confirmatory factor analysis (CFA): standardized loadings and residual variances by language group (WLSMV estimator, prespecified five-factor model).

Item	Domain	Russian loading	Russian residual var.	Kazakh loading	Kazakh residual var.	Flag
item1	Information	0.890	0.208	0.877	0.231	
item2	Information	0.881	0.223	0.931	0.133	
item3	Information	**0.958**	0.082	0.942	0.112	**≥.95**
item4	Comprehensive Care	0.921	0.151	0.874	0.235	
item5	Comprehensive Care	0.910	0.171	0.944	0.109	
item6	Comprehensive Care	0.872	0.240	0.823	0.323	
item7	Comprehensive Care	0.923	0.149	0.878	0.230	
item8	Comprehensive Care	0.889	0.210	0.929	0.137	
item9	Comprehensive Care	0.846	0.284	0.899	0.193	
item10	Quality of Life	**0.977**	0.046	**0.998**	0.004	**≥.95**

item11	Quality of Life	**0.952**	0.094	**0.985**	0.030	**≥.95**
item12	Quality of Life	0.789	0.377	0.813	0.339	
item13	Quality of Life	0.547	0.701	0.547	0.701	
item14	Quality of Life	0.820	0.327	**0.953**	0.091	**≥.95**
item15	Quality of Life	0.942	0.113	**0.969**	0.062	**≥.95**
item16	Quality of Life	**1.000**	**-0.001**	**0.966**	0.067	**Heywood case (RU)**
item17	Quality of Life	**0.989**	0.021	**0.975**	0.050	**≥.95**
item18	Quality of Life	0.891	0.206	0.902	0.187	
item19	Relationships	0.900	0.190	**0.989**	0.022	**≥.95**
item20	Relationships	0.931	0.133	**0.995**	0.009	**≥.95**
item21	Relationships	0.889	0.210	0.945	0.107	
item22	Relationships	0.700	0.510	0.857	0.265	
item23	Relationships	0.743	0.448	0.862	0.256	
item24	Relationships	0.893	0.203	0.853	0.272	
item25	Relationships	0.869	0.244	0.878	0.229	
item26	Relationships	0.798	0.363	0.867	0.249	
item27	Relationships	0.668	0.553	0.788	0.379	
item28	Relationships	0.731	0.465	0.886	0.215	
item29	Existential Survivorship	0.944	0.108	0.929	0.137	
item30	Existential Survivorship	0.920	0.154	0.937	0.123	
item31	Existential Survivorship	0.884	0.219	0.947	0.103	
item32	Existential Survivorship	0.929	0.136	0.947	0.104	
item33	Existential Survivorship	0.903	0.184	0.944	0.109	
item34	Existential Survivorship	0.939	0.117	0.924	0.147	
item35	Existential Survivorship	**0.953**	0.093	0.883	0.220	**≥.95**

Bold loadings indicate values ≥ .95. Negative residual variance for item16 (Russian sample) indicates a Heywood case. All loadings significant at p < .001. Estimator: WLSMV, polychoric correlations, ordered-categorical items (0–3). Factor correlations for this model are reported in Table 3b of the main text.

Table S5. CFA model convergence and warnings by language group (baseline model, no residual covariance).

Language	N	χ² (scaled)	df	CFI	TLI	RMSEA	SRMR	Model warnings
Russian	201	1570.996	550	0.975	0.973	0.096	0.116	Negative residual variance for item16 (Heywood case); polychoric(item16,item17) ≈ 1.0; non-positive-definite parameter covariance matrix (smallest eigenvalue = -3.18×10^{-17})
Kazakh	203	1708.544	550	0.973	0.971	0.102	0.099	Non-positive-definite parameter covariance matrix (smallest eigenvalue = -5.60×10^{-17}); no negative residual variances; eight items with loadings ≥ .95

Sensitivity analyses addressing the item16–item17 local dependence (residual covariance vs. item removal) are reported in Table 3 of the main text.

Table S6. Category frequencies for all 35 CaSUN items, by language group

Item	Russian (n=201)				Kazakh (n=203)			
	0 (no need)	1 (met)	2 (weak unmet)	3 (strong unmet)	0 (no need)	1 (met)	2 (weak unmet)	3 (strong unmet)
Item 1	53	48	77	23	43	38	88	34
Item 2	52	47	87	15	47	50	86	20
Item 3	58	39	76	28	40	58	66	39
Item 4	54	40	73	34	44	43	70	46
Item 5	51	45	79	26	42	52	70	39
Item 6	47	52	80	22	37	52	74	40
Item 7	54	49	70	28	43	54	71	35
Item 8	53	66	61	21	48	55	66	34
Item 9	57	66	55	23	46	61	50	46
Item 10 *	54	68	70	**9**	55	30	97	21
Item 11 *	65	59	68	**9**	61	31	76	35
Item 12 *	69	74	51	**7**	64	54	69	16
Item 13 *	121	52	23	**5**	141	28	29	**5**
Item 14	113	41	33	14	105	33	36	29
Item 15	120	50	20	11	106	33	40	24
Item 16	123	49	19	10	110	39	29	25
Item 17	129	43	18	11	110	42	28	23
Item 18 *	120	42	32	**7**	95	42	36	30
Item 19	64	57	70	10	53	32	84	34
Item 20	63	56	57	25	50	38	67	48
Item 21	73	65	53	10	57	51	55	40
Item 22 *	70	85	40	**6**	71	39	70	23
Item 23 *	88	71	35	**7**	78	38	64	23
Item 24 *	87	60	52	**2**	66	42	68	27
Item 25 *	99	57	39	**6**	64	72	47	20
Item 26 *	88	65	43	**5**	65	70	48	20
Item 27 *	136	48	16	**1**	100	55	27	21
Item 28 *	115	55	28	**3**	82	56	42	23
Item 29	68	59	61	13	72	34	61	36
Item 30	66	60	58	17	71	31	63	38
Item 31	69	60	61	11	66	50	50	37
Item 32	75	53	60	13	61	52	57	33

Item 33	71	62	57	11	55	65	55	28
Item 34	73	64	41	23	59	57	54	33
Item 35	72	67	36	26	63	44	48	48

** Denotes items with a category cell n < 10 in at least one language group, flagged as potentially sparse for polychoric threshold estimation. In the Russian sample, the weakest cell was item27 (strong unmet need, n=1); the Kazakh sample showed no cell below n=5 (item13 only).*
